# Some notes on Sonine–Gegenbauer integrals

**DOI:** 10.1080/10652469.2018.1538215

**Published:** 2018-10-29

**Authors:** Peter Grandits

**Affiliations:** Institut für Stochastik und Wirtschaftsmathematik, TU Wien, Vienna, Austria

**Keywords:** Integrals of Bessel functions, Sonine–Gegenbauer-type integrals, Green's functions, 33C10, 35C15

## Abstract

We provide an explicit formula for a Sonine–Gegenbauer integral, which seems to be unknown in the literature so far. For another type of these integrals, we show a dependence relation over the rational functions, including the explicit calculation of the coefficient functions.

## 1. Introduction

In this note, we want to give some explicit formulas for, respectively, dependence relations between some Sonine–Gegenbauer integrals for special parameter values, which we did not find in the existing literature. More precisely, we are interested in integrals involving modified Bessel functions of the second kind and the exponential function, i.e. we give an explicit formula for the integral

$$\int_{0}^{\infty }e^{-\xi }\frac{K_{1}\left(\sqrt{\xi^{2}+x^{2}}\right)x}{\sqrt{\xi^{2}+x^{2}}}d\xi ,$$

see Section 2, and we show a dependence relation over the field of rational functions for the Sonine–Gegenbauer integrals

$$\gamma_{l}(x):=\int_{0}^{\infty }e^{-\xi }K_{0}\left(\sqrt{2\xi^{2}+2x^{2}}\right)\xi^{l}\;d\xi ,$$

for *l*=0,1,2,3, see Section 3. Moreover, we explicitly provide the rational coefficient functions there.

In the existing literature, explicit formulas for

$$\int_{0}^{\infty }xK_{0}\left[\alpha \sqrt{x^{2}+\beta^{2}}\right]\sin(xy)\;dx,$$

resp. for

$$\int_{0}^{\infty }K_{0}\left[\alpha \sqrt{x^{2}+\beta^{2}}\right]\cos(xy)\;dx,$$

are known: see e.g. [1, p.112(42) and p.56(43)] or [2, 6.677/5]. Moreover, one can also take a look at [3, 13.4.3(3)], which includes the two formulas above as special limiting cases.

Replacing the modified Bessel function $K_{0}$ by the Bessel functions $J_{0}$ and $Y_{0}$, one finds expressions for integrals of the type above in [[4, Section 4],[5, Section 4]].

Let us finally mention the papers [6,7], where different types of integrals over the Bessel function are considered.

We shall use for the proofs in this paper PDE methods, basically Green's formula for value functions appearing in stochastic control theory.

## 2. A first Sonine–Gegenbauer integral

In this section, we give an explicit formula for a certain Sonine–Gegenbauer integral in terms of some special functions. We shall denote here and in the sequel $K_{\alpha }(x)$ as the modified Bessel function of the second kind of order *α* and $L_{\alpha }(x)$ as the Struve L function of order *α*. We provide the following lemma.

Lemma 2.1 One has for *x*>0

$$\int_{0}^{\infty }e^{-\xi }\frac{K_{1}\left(\sqrt{\xi^{2}+x^{2}}\right)x}{\sqrt{\xi^{2}+x^{2}}}d\xi =\frac{\pi }{2}-xK_{0}(x)-\frac{\pi x}{2}\left(K_{0}(x)L_{1}(x)+K_{1}(x)L_{0}(x)\right).$$

Proof. We consider on the set G:={(x,y)|0<x<y} the following boundary value problem

(2.1) $$\begin{array}{rl} & LV:=\frac{1}{2}V_{x}+\frac{1}{2}V_{y}+\frac{1}{2}\Delta V=0,\\ & V(0,y)=0,\\ & V(x,\infty )=1-e^{-x},\\ & V_{x}(t,t)=V_{y}(t,t),\;t>0. \end{array}$$

This system has the solution $V(x,y)=1-e^{-x}-e^{-y}+e^{-x-y}=(1-e^{-x})(1-e^{-y})$. V(x,y) has the probabilistic interpretation as the probability that a two-dimensional Brownian motion with drift 1/2 in *x* – as well as in *y* – direction does not exit the positive quadrant, if it starts at (x,y).

Performing the transformation $w(x,y):=(V(x,y)-(1-e^{-x}))\;e^{\left(x+y\right)/2}$ gives the function $w(x,y)=-e^{\left(x-y\right)/2}+e^{\left(-x-y\right)/2}$, which solves the system

(2.2) $$\begin{array}{rl} & \Delta w-\frac{1}{2}w=0,\\ & w(0,y)=0,\\ & w(x,\infty )=0,\\ & w_{\nu }(t,t)=-\frac{1}{\sqrt{2}},\;t>0, \end{array}$$

where $w_{\nu }$ denotes the normal derivative in the direction $\nu :=\frac{1}{\sqrt{2}}(1,-1)$. The first equation is a so-called modified Helmholtz equation and has $\left(1/2\pi )K_{0}(\rho |(x,y)|\right)$ as the fundamental solution. We use here and in the sequel (for x≥0, y≥0, ξ≥0, η≥0) the definitions $\rho :=\sqrt{\frac{1}{2}}$, $\vec{x}:=(x,y)$, ${\vec{x}}^{m}:=(-x,y)$, $\vec{\xi }:=(\xi ,\eta )$, as well as

(2.3) $$E(\vec{\xi };\vec{x}):=\frac{1}{2\pi }\left(K_{0}\left(\rho \left|\vec{\xi }-\vec{x}\right|\right)-K_{0}\left(\rho \left|\vec{\xi }-{\vec{x}}^{m}\right|\right)\right),$$

for Green's function, which is odd w.r.t. the variable *x*. We specialize now $\vec{x}$ to be a point on the boundary of *G*, namely (x,x), with *x*>0. Green's second fundamental theorem gives

(2.4) $$\frac{w(x,x)}{2}=\int_{\partial G}\left[E(\vec{\xi };(x,x))\frac{\partial w}{\partial \nu }(\vec{\xi })-w(\vec{\xi })\frac{\partial E}{\partial \nu }(\vec{\xi };(x,x))\right]dS(\vec{\xi }).$$

One comment on this formula is in order. Green's formula is usually proved for bounded domains with smooth boundary. So it holds for a triangle domain with corners (0,0),(0,n),(n,n), where the corners are smoothed. Going to the limit gives Equation (2.4) without problems, because on the segment (x,n), 0<x<n, the integrand is exponentially small in *n*, whereas its length is only *n*. Moreover, at the smoothed corners the integrand is bounded, since *x*>0 is assumed.

Using the fact that Green's function *E* as well as the function *w* vanishes on the *y*-axis by construction, we arrive at

(2.5) $$\frac{M(x)}{2}=\int_{0}^{\infty }\left[E(\xi ,\xi ;x,x)\left(-\frac{1}{\sqrt{2}}\right)-M(\xi )\frac{\partial E}{\partial \nu }(\xi ,\xi ;x,x)\right]dS(\xi ),$$

where we have written M(x) for w(x,x) and used the fourth equation of Equation (2.2). Calculating the normal derivative and employing $M(x)=-1+e^{-x}$, we end up with

(2.6) $$\begin{array}{rl} \frac{-1+e^{-x}}{2} & =\frac{1}{2\pi }\int_{0}^{\infty }\left[K_{0}\left(\sqrt{\xi^{2}+x^{2}}\right)-K_{0}\left(|\xi -x|\right)\right.\\ & \;+\left.(1-e^{-\xi })\frac{K_{1}\left(\sqrt{\xi^{2}+x^{2}}\right)x}{\sqrt{\xi^{2}+x^{2}}}\right]d\xi , \end{array}$$

where we have used the fact that $K_{0}^{\prime }(x)=-K_{1}(x)$.

To evaluate $\int_{0}^{\infty }K_{0}(\sqrt{\xi^{2}+x^{2}})\;d\xi$, we use that $K_{0}(\sqrt{\xi^{2}+x^{2}})$ is basically the density of a symmetric generalized hyperbolic distribution for certain parameters (see e.g. [8, p.321]). The normalization condition provides (see also [3, 13.4.3(6)])

(2.7) $$\int_{0}^{\infty }K_{0}\left(\sqrt{\xi^{2}+x^{2}}\right)d\xi =\sqrt{\frac{x\pi }{2}}K_{1/2}(x)=\frac{\pi }{2}e^{-x}.$$

Moreover, one knows (see [2, 6.561/4] and the well-known relation $L_{-1}(x)-L_{1}(x)=2/\pi )$ that

(2.8) $$\int_{0}^{\infty }K_{0}\left(|\xi -x|\right)d\xi =\frac{\pi }{2}+xK_{0}(x)+\frac{\pi x}{2}\left(K_{0}(x)L_{1}(x)+K_{1}(x)L_{0}(x)\right),$$

as well as, see [3, 13.4.3(6)],

(2.9) $$\int_{0}^{\infty }\frac{K_{1}\left(\sqrt{\xi^{2}+x^{2}}\right)x}{\sqrt{\xi^{2}+x^{2}}}d\xi =\frac{\pi \;e^{-x}}{2}.$$

hold. Plugging Equations (2.7), (2.8) and (2.9) into Equation (2.6), gives, after some rearrangement, the assertion of the lemma.

## 3. A dependence relation between some Sonine–Gegenbauer integrals

Once the coefficients *a* and *b* in $e^{-a\xi }K_{0}(b\sqrt{\xi^{2}+x^{2}})$ do *not* coincide, the calculation of the corresponding integrals becomes more complicated. This seems to be in accordance with the case $e^{-a\xi }K_{0}(b\xi )$, see [3, Chapter V]. We are interested in this section in the case $b/a=\sqrt{2}$, (w.l.o.g. we restrict here to *a*=1, $b=\sqrt{2}$) since this is the case relevant in the optimal control problem, which motivated our work (see the last section of this paper). The object of our interest are the Sonine–Gegenbauer integrals

(3.1) $$\gamma_{l}(x):=\int_{0}^{\infty }e^{-\xi }K_{0}\left(\sqrt{2\xi^{2}+2x^{2}}\right)\xi^{l}\;d\xi .$$

Our main result will be that the functions $\gamma_{0}$, $\gamma_{1}$, $\gamma_{2}$, $\gamma_{3}$, $K_{0}(\sqrt{2}x)$ and $K_{1}(\sqrt{2}x)$ are not independent over the field of rational functions, which comes, at least to the author, as a surprise. More precisely, we have

Theorem 3.1 There exist rational functions $p_{0}(x),$$p_{1}(x),$$p_{2}(x),$$p_{3}(x),$$q_{0}(x)$ and $q_{1}(x),$ s.t.

$$\sum_{l=0}^{3}p_{l}(x)\gamma_{l}(x)+q_{0}(x)K_{0}(\sqrt{2}x)+q_{1}(x)K_{1}(\sqrt{2}x)=0,$$

for x>0, with the following coefficient functions

$$\begin{array}{rl} p_{0}(x) & :=-\frac{3}{x^{3}}+\frac{6}{x^{2}}-\frac{5}{x}-11+x,\\ p_{1}(x) & :=\frac{4}{x^{5}}-\frac{8}{x^{4}}+\frac{5}{x^{3}}+\frac{18}{x^{2}}-\frac{2}{x}+3,\\ p_{2}(x) & :=-\frac{5}{x^{5}}+\frac{10}{x^{4}}-\frac{11}{x^{3}}-\frac{13}{x^{2}}-\frac{1}{x},\\ p_{3}(x) & :=-\frac{1}{x^{5}}+\frac{2}{x^{4}}-\frac{2}{x^{3}}-\frac{3}{x^{2}},\\ q_{0}(x) & :=\frac{2}{x^{3}}-\frac{4}{x^{2}}+\frac{3}{x}+8-x,\\ q_{1}(x) & :=\sqrt{2}\left(\frac{1}{x^{2}}-\frac{2}{x}+1\right). \end{array}$$

We have used in Section 2 Green's theorem and the knowledge of a certain explicit solution for a boundary value problem involving the modified Helmholtz equation (which had a certain probabilistic interpretation) to calculate a certain Sonine–Gegenbauer integral. Here we take as starting point the following system:

(3.2) $$\begin{array}{rl} & V_{x}+\frac{1}{2}\Delta V=0,\\ & V(0,y)=0,\\ & V(x,\infty )=1-e^{-2x},\\ & V_{x}(t,t)=V_{y}(t,t),\;t>0, \end{array}$$

on the set *G*, defined in Section 2. This system stems from an optimal control problem with value function V(x,y), see [9], and has as solution $V(x,y)=1-e^{-2x}-2x\;e^{-x-y}$. The transformation $w(x,y)=e^{x}(V(x,y)-(1-e^{-2x}))$, and using Green's formula in exactly the same way as in Section 2 yields the following equation

(3.3) $$\begin{array}{rl} -x\;e^{-x} & =\frac{1}{2\pi }\int_{0}^{\infty }\left[K_{0}\left(\sqrt{2}|\xi -x|\right)-K_{0}\left(\sqrt{2\xi^{2}+2x^{2}}\right)\right]\left(-2\xi \;e^{-\xi }-2\;e^{-\xi }\right)d\xi \\ & \;+\frac{2x}{\pi }\int_{0}^{\infty }\frac{\xi \;e^{-\xi }K_{1}\left(\sqrt{2\xi^{2}+2x^{2}}\right)}{\sqrt{2\xi^{2}+2x^{2}}}d\xi . \end{array}$$

We start with the following simple lemma.

Lemma 3.1 The Sonine–Gegenbauer integrals $\gamma_{0}$ and $\gamma_{1}$ fulfil

$$\gamma_{1}(x)+\gamma_{0}(x)-x\left(\gamma_{0}(x)-K_{0}(\sqrt{2}x)\right)=G(x),$$

with

$$\begin{array}{rl} G(x) & :=(x+1)\;e^{-x}\int_{0}^{x}K_{0}(\sqrt{2}z)\;e^{z}\;dz\\ & \;-e^{-x}\int_{0}^{x}K_{0}(\sqrt{2}z)\;e^{z}z\;dz+(x+1)\;e^{-x}\int_{0}^{\infty }K_{0}(\sqrt{2}z)\;e^{-z}\;dz\\ & \;+e^{-x}\int_{0}^{\infty }K_{0}(\sqrt{2}z)\;e^{-z}z\;dz-\pi x\;e^{-x}. \end{array}$$

Proof. The last term in Equation (3.3) can be written as $-(1/\pi )\gamma_{1}^{\prime }(x)$. Moreover, simple calculation yields

$$\begin{array}{rl} \frac{1}{2\pi }\int_{0}^{\infty }K_{0}\left(\sqrt{2}|\xi -x|\right)(-2\xi -2)\;e^{-\xi }\;d\xi & =\frac{1}{2\pi }\left((-2x-2)\;e^{-x}\int_{0}^{x}K_{0}(\sqrt{2}z)\;e^{z}\;dz\right.\\ & \;\left.+2\;e^{-x}\int_{0}^{x}K_{0}(\sqrt{2}z)\;e^{z}z\;dz\right.\\ & \;\left.+(-2x-2)\;e^{-x}\int_{0}^{\infty }K_{0}(\sqrt{2}z)\;e^{-z}\;dz\right.\\ & \;\left.-2\;e^{-x}\int_{0}^{\infty }K_{0}(\sqrt{2}z)\;e^{-z}z\;dz\right). \end{array}$$

Hence, multiplying by *π*, Equation (3.3) can be written as

(3.4) $$\gamma_{1}(x)+\gamma_{0}(x)-\gamma_{1}^{\prime }(x)=G(x).$$

Finally, integration by parts gives

(3.5) $$\gamma_{1}^{\prime }(x)=x\int_{0}^{\infty }\frac{K_{0}^{\prime }(\sqrt{2\xi^{2}+2x^{2}})2\xi }{\sqrt{2\xi^{2}+2x^{2}}}e^{-\xi }\;d\xi =x\left(-K_{0}(\sqrt{2}x)+\gamma_{0}(x)\right).$$

Equations (3.4) and (3.5) prove the lemma.

We come now to the proof of our main result.

Proof **Proof of Theorem 3.1**

Let *x*>0. By continuity it suffices to show

(3.6) $$\gamma_{3}+f_{2}\gamma_{2}+f_{1}\gamma_{1}+f_{0}\gamma_{0}+r_{0}K_{0}(\sqrt{2}x)+r_{1}K_{1}(\sqrt{2}x)=0,$$

with

$$f_{2}(x):=\frac{p_{2}(x)}{p_{3}(x)},\;f_{1}(x):=\frac{p_{1}(x)}{p_{3}(x)},\;f_{0}(x):=\frac{p_{0}(x)}{p_{3}(x)},\;r_{0}(x):=\frac{q_{0}(x)}{p_{3}(x)},\;r_{1}(x):=\frac{q_{1}(x)}{p_{3}(x)},$$

and by Lemma 3.1, we need to show

(3.7) $$\begin{array}{rl} & \gamma_{3}+f_{2}\gamma_{2}+\gamma_{1}\left(f_{1}+\frac{f_{0}}{x-1}\right)+f_{0}K_{0}(\sqrt{2}x)\frac{x}{x-1}+r_{0}K_{0}(\sqrt{2}x)\\ & \;+r_{1}K_{1}(\sqrt{2}x)-\frac{Gf_{0}}{x-1}=0. \end{array}$$

Defining now

$$g_{2}(x):=f_{2}(x),\;g_{1}(x):=f_{1}(x)+\frac{f_{0}(x)}{x-1},$$

and

$$F(x):=f_{0}(x)K_{0}(\sqrt{2}x)\frac{x}{x-1}+r_{0}(x)K_{0}(\sqrt{2}x)+r_{1}(x)K_{1}(\sqrt{2}x)-\frac{G(x)f_{0}(x)}{x-1},$$

we arrive at

(3.8) $$\gamma_{3}+g_{2}\gamma_{2}+\gamma_{1}g_{1}+F=0,$$

which is to be shown. Since the l.h.s. of Equation (3.8) tends to zero for x→∞ (the $\gamma_{i}$'s and *F* do so exponentially!!), it is sufficient to show that the derivative of the l.h.s. is identically zero, i.e. we have to show

(3.9) $$\gamma_{3}^{\prime }+g_{2}\gamma_{2}^{\prime }+g_{2}^{\prime }\gamma_{2}+\gamma_{1}^{\prime }g_{1}+\gamma_{1}g_{1}^{\prime }+F^{\prime }=0.$$

By the same integration by parts as in the proof of Lemma 3.1 one has for l≥1

(3.10) $$\gamma_{l}^{\prime }=x\left(-1_{\left\{l=1\right\}}K_{0}(\sqrt{2}x)+\gamma_{l-1}-(l-1)\gamma_{l-2}\right),$$

where 1 denotes the indicator function. (Note that the last term vanishes for *l*=1, so there is no need to define $\gamma_{-1}$ .) Plugging this into Equation (3.9) provides

(3.11) $$\gamma_{2}\left(x+g_{2}^{\prime }\right)+\gamma_{1}\left(-2x+xg_{2}+g_{1}^{\prime }\right)+\gamma_{0}\left(-xg_{2}+xg_{1}\right)-xg_{1}K_{0}(\sqrt{2}x)+F^{\prime }=0.$$

Again, by continuity it suffices to show

(3.12) $$\gamma_{2}+\gamma_{1}\frac{-2x+xg_{2}+g_{1}^{\prime }}{x+g_{2}^{\prime }}+\gamma_{0}\frac{-xg_{2}+xg_{1}}{x+g_{2}^{\prime }}-\frac{xg_{1}K_{0}(\sqrt{2}x)}{x+g_{2}^{\prime }}+\frac{F^{\prime }}{x+g_{2}^{\prime }}=0.$$

Using once more Lemma 3.1, we have to show

(3.13) $$\begin{array}{rl} & \gamma_{2}+\gamma_{1}\left[\frac{-2x+xg_{2}+g_{1}^{\prime }}{x+g_{2}^{\prime }}+\frac{xg_{1}-xg_{2}}{\left(x-1)(x+g_{2}^{\prime }\right)}\right]\\ & \;+\left(\frac{x}{x-1}K_{0}(\sqrt{2}x)-\frac{G}{x-1}\right)\left(\frac{-xg_{2}+xg_{1}}{x+g_{2}^{\prime }}\right)-\frac{xg_{1}K_{0}(\sqrt{2}x)}{x+g_{2}^{\prime }}+\frac{F^{\prime }}{x+g_{2}^{\prime }}=0. \end{array}$$

Denoting the first [⋯] by $h_{1}(x)$ and the second line of Equation (3.13) by H(x), we have to show that

(3.14) $$\gamma_{2}(x)+\gamma_{1}(x)h_{1}(x)+H(x)=0,$$

holds.

Again, the l.h.s. of Equation (3.14) tends to zero for x→∞, so it suffices to show that its derivative is identically zero, i.e. that

(3.15) $$\gamma_{2}^{\prime }+\gamma_{1}^{\prime }(x)h_{1}(x)+\gamma_{1}(x)h_{1}^{\prime }(x)+H^{\prime }(x)=0,$$

or

(3.16) $$x(\gamma_{1}-\gamma_{0})+x(\gamma_{0}-K_{0}(\sqrt{2}x))h_{1}+\gamma_{1}h_{1}^{\prime }+H^{\prime }=0,$$

hold. By Lemma 3.1, this is equivalent to

(3.17) $$\begin{array}{rl} & \gamma_{1}\left(x^{2}-2x+xh_{1}+(x-1)h_{1}^{\prime }\right)+K_{0}(\sqrt{2}x)\left(-x^{2}+xh_{1}\right)\\ & \;-G(x)\left(xh_{1}-x\right)+(x-1)H^{\prime }=0. \end{array}$$

Now, it can be checked by simple calculation (symbolic computation, e.g. by MAPLE, is helpful!) that

(3.18) $$x^{2}-2x+xh_{1}+(x-1)h_{1}^{\prime }=0$$

for *x*>0 holds.

Moreover, again using symbolic calculation, we find

(3.19) $$\begin{array}{rl} & K_{0}(\sqrt{2}x)\left(-x^{2}+xh_{1}\right)-G(x)\left(xh_{1}-x\right)+(x-1)H^{\prime }\\ & \;=q(x)\left\{\sqrt{2}xK_{1}(\sqrt{2}x)+xK_{0}(\sqrt{2}x)\right.\\ & \;+\left.e^{-x}\int_{0}^{x}K_{0}(\sqrt{2}z)\;e^{z}z\;dz-e^{-x}\int_{0}^{x}K_{0}(\sqrt{2}z)\;e^{z}\;dz-e^{-x}\right\}, \end{array}$$

for some rational function q(x). Denoting the curly bracket in Equation (3.19) by s(x), we prove now that s(x)=0 for all *x*.

We first check that $s(0)=\lim_{x\to 0}\sqrt{2}xK_{1}(\sqrt{2}x)-1=0$.

Let $t(x):=e^{x}s(x)$ and note that clearly t(0)=0 holds. Using the well-known formula $K_{1}^{\prime }(z)=-K_{0}(z)-K_{1}(z)/z$, it is easy to check that $t^{\prime }(x)=0$ for all *x*, hence t(x)=0 and s(x)=0. This implies that the expression in Equation  (3.19) is identically zero, which shows together with Equation (3.18) that Equation (3.17) is true, which proves the theorem.

## 4. Concluding remarks and open problems

In this last chapter we want to indicate, how we found the equation of Theorem 3.1, and the correct coefficient functions $p_{i},q_{i}$. Furthermore, we pose some open problems.

As noted earlier, this work had its origin in an optimal control problem. As explained in [9], the aim in this problem is to maximize the probability that both of two companies survive. The prerequisites (for details see [9]) are that the wealth of these companies are described by Brownian motion with drift and that the controller can support them by distributing a positive drift (adding up to one) to these companies. As mentioned in Section 3, McKean and Shepp found an explicit solution for this problem, i.e. they found an explicit formula for the value function and the optimal strategy. An assumption of their paper was that the wealth processes of these companies have equal volatilities (set there equal to 1). For different volatilities the problem was open. In [10] we assume that the volatilities are almost equal, i.e. $\sigma_{1}^{2}=1,\;\sigma_{2}^{2}=1+\delta$ with *δ* small, and try to solve the problem approximately by linearization.

It turns out that there is a free boundary curve, where one should change from giving the whole positive drift to one company to giving it to the other one. Say this curve is denoted by b(x)=x+η(x) (b(x)=x is the solution in case of equal volatilities). One finds that the function η(x) fulfils an integral equation of the form

$$\kappa (x)\eta (x)+\frac{2}{\pi }\int_{0}^{\infty }\left[K_{0}(\sqrt{2}|\xi -x|)-K_{0}(\sqrt{2}\sqrt{\xi^{2}+x^{2}})\right]e^{-\xi }\eta (\xi )\;d\xi +f(x)=0,$$

for some known function f(x). Calculating κ(x) we found

$$\begin{array}{rl} \kappa (x) & =-\frac{1}{2\pi }\int_{0}^{\infty }\frac{K_{0}(\sqrt{2\xi^{2}+2x^{2}})\;e^{-\xi }}{2\xi^{2}+2x^{2}}\left(8\xi^{2}x\right)d\xi \\ & \;+\frac{1}{2\pi }\int_{0}^{\infty }\frac{K_{0}^{\prime }(\sqrt{2\xi^{2}+2x^{2}})\;e^{-\xi }}{(2\xi^{2}+2x^{2}{)}^{3/2}}\left(16\xi^{2}x+8\xi x^{2}+\cdots \right)d\xi \\ & \;-\frac{\sqrt{2}\;e^{-x}}{\pi }\int_{0}^{x}K_{0}^{\prime }(\sqrt{2}s)\;e^{s}\frac{x-s}{s}+\frac{\sqrt{2}x}{2s^{2}}+\frac{\sqrt{2}(x-1)}{2s}ds\\ & \;-\frac{\sqrt{2}\;e^{-x}}{\pi }\int_{0}^{x}K_{0}^{\prime }(\sqrt{2}s)\;e^{-s}\frac{x+s}{s}+\frac{\sqrt{2}x}{2s^{2}}-\frac{\sqrt{2}(x-1)}{2s}ds\\ & \;-\frac{\sqrt{2}\;e^{-x}}{\pi }\int_{x}^{\infty }K_{0}^{\prime }(\sqrt{2}s)\;e^{-s}\frac{x+s}{s}ds\\ & \;-\frac{2}{\pi }e^{-x}+e^{-x}(x+1), \end{array}$$

where +⋯ denotes some similar terms. To get an idea of the behaviour of the function κ(x), we evaluated it numerically, and to our surprise found out that it seems to be identically equal to zero. So in order to proceed, we had to prove that this holds rigorously, because this makes out the difference between a Fredholm integral equation of the first and one of the second kind, which have different solution behaviour.

Let us sketch now very briefly, how we came from the equation κ≡0 to the dependence relation in Theorem 3.1. We noted that the terms beside the integrals, i.e. $\kappa_{1}(x):=-(2/\pi )\;e^{-x}+e^{-x}(x+1)$ satisfy $L\kappa_{1}(x)\equiv 0$, with $L:=d^{2}/dx^{2}+2(d/dx)+1$. So, if κ≡0 is indeed true, we should have $L(\kappa (x)-\kappa_{1}(x))\equiv 0$. After a very lengthy but elementary calculation, this equation provided the equation of Theorem 3.1.

*Some open problems* :

Although we have proven Theorem 3.1 rigorously, the proof gives not much insight, since the correct coefficient functions ‘fell more or less from heaven’. One could say that their discovery was only possible by coincidence, namely by observing numerically that a certain function seems to be identically equal to zero.

As this is clearly not satisfying, this paper should also stimulate future research. Possible questions are:

1. Can we find the coefficient functions $p_{i}$ and $q_{i}$ in a systematic way? One obvious try would be to roll up the recursive procedure in the proof of Theorem 3.1, and to find determining equations for the coefficient functions. Unfortunately, the resulting equations are far too complicated to be solved (if one does not know the solution and can verify it by plugging in).
2. Is the result, which we have proved for *fourγ*'s, true for other numbers?
3. Is it possible to prove the result for values of *a* and *bnot* satisfying $b/a=\sqrt{2}$?
